# Relationship between Particulate Matter (PM_10_) and Airway Inflammation Measured with Exhaled Nitric Oxide Test in Seoul, Korea

**DOI:** 10.1155/2020/1823405

**Published:** 2020-03-17

**Authors:** Juwhan Choi, Jae Kyeom Sim, Jee Youn Oh, Young Seok Lee, Gyu Young Hur, Sung Yong Lee, Jae Jeong Shim, Ji-yong Moon, Kyung Hoon Min

**Affiliations:** ^1^Division of Pulmonary, Allergy and Critical Care Medicine, Department of Internal Medicine, Korea University Guro Hospital, Korea University College of Medicine, Seoul 08308, Republic of Korea; ^2^Division of Pulmonary, Allergy and Critical Care Medicine, Department of Internal Medicine, Hanyang University Guri Hospital, Hanyang University College of Medicine, Gyeongchun-ro 153, Guri-si, Gyeonggi-do 11923, Republic of Korea

## Abstract

**Purpose:**

Particulate matter (PM) is increasing every year in Asia. It is not fully understood how the airway is affected when inhaling PM. We investigated the correlation between particulate matter with a diameter of less than 10 *μ*m (PM_10_) and fractional exhaled nitric oxide (FeNO) to determine whether PM causes airway inflammation. *Material and Methods*. We analyzed patients who visited our outpatient clinic and tested FeNO from January 2016 to December 2017 at the Korea University Guro Hospital. PM_10_ data were provided by the government of the Republic of South Korea, and measuring station of PM_10_ is located 800 meters from the hospital. We analyzed the correlation between PM_10_ and FeNO by a Pearson correlation analysis and by a multivariate linear regression analysis. To identify the most correlated times, we analyzed the correlation between the FeNO and PM_10_ daily average from the day of visit to 4 days before visit.

**Results:**

FeNO positively correlated with PM_10_ at two days before hospital visit in the Pearson correlation (Pearson correlation coefficient = 0.057; *P*-value = 0.023) and in the multivariate linear regression analysis (*B* = 0.051, *P*-value = 0.026). If the PM_10_ increased by 100 *μ*g/m^3^, the FeNO result was expected to rise to 8.3 ppb in healthy people without respiratory disease.

**Conclusion:**

The positive correlation was found in both healthy people and asthmatic patients. Therefore, PM_10_ can increase airway inflammation.

## 1. Introduction

Particulate matter (PM) is a global environmental issue [1]. Recently, PM is increasing every year in China and in neighboring countries [2]. Particularly in Korea, outdoor activities are increasingly difficult and quality of life is diminishing from PM [3]. The higher the PM levels, the more people complain of various respiratory symptoms, and even those who do not have respiratory diseases need medical treatment reducing respiratory symptoms [4].

Generally, PM is known to cause abnormal inflammatory and coagulation responses in the entire body [5, 6]. And, PM can act as a direct irritant to the airway and causes respiratory disease. Incidences of airway disease and the frequency of acute exacerbations of asthma or chronic obstructive pulmonary disease (COPD) are increasing [7]. In addition, incidences of lung cancer are also expected to increase [8]. However, it is not fully understood how the airway reacts when inhaling PM. In addition, there is a lack of research on the adverse effects of PM in healthy people without respiratory disease.

Among the known respiratory tests, fractional exhaled nitric oxide (FeNO) is one that is a marker of airway inflammation [9]. FeNO is useful because it is noninvasive and can be easily examined in an outpatient clinic. When treating asthma and COPD, FeNO can predict airway inflammation and is also used to predict inhaled corticosteroid (ICS) responses [10]. Therefore, we investigated the correlation between PM and FeNO to determine whether PM causes airway inflammation.

## 2. Materials and Methods

### 2.1. Data Collection

We obtained FeNO results from January 2016 to December 2017 at the Korea University Guro Hospital by searching the hospital's electronic records. FeNO was measured using the nitric oxide delivery system monitor approved by the US Food and Drug Administration (NIOX MINO, Aerocrine, Sweden). We asked subjects about drugs used which could affect FeNO result at the time of the test. We instruct subjects to avoid exercise, smoking, and caffeine ingestion within 1 hour of FeNO test. This study was approved by the Institutional Review Board of the Korea University Guro Hospital (approval number: K2018-0377-001). Since our study was retrospective, patient consent was not necessary, and we maintained patient confidentiality.

We collected and analyzed the following medical records: age, gender, history of respiratory disease, pulmonary related treatment before the outpatient clinic visit, and the pulmonary function test (PFT).

We used particulate matter with a diameter of less than 10 *μ*m (PM_10_) as the representative value. PM_10_ data were provided by the government of the Republic of South Korea. PM_10_ data were obtained from a government measuring station located 800 meters from the Korea University Guro Hospital. And humidity and temperature data were also provided by the government of the Republic of South Korea.

### 2.2. Subjects

We included patients who visited our outpatient clinic for the first time and those who visited periodically due to asthma, allergic rhinitis, and COPD. Asthma was diagnosed based on the Global Initiative for Asthma (GINA) guidelines (patients showed positive for the bronchodilator reversibility test, positive for the bronchial challenge test, or positive for the exercise challenge test) [11]. Allergic rhinitis was diagnosed based on the Allergic Rhinitis and its Impact on Asthma (ARIA) guideline (patients had a typical history of allergic symptoms, such as rhinorrhea, sneezing, sneezing, nasal obstruction, and pruritus and diagnostic tests such as allergen-specific immunoglobulin E in the skin or blood specific immunoglobulin E) [12]. COPD was diagnosed based on the Global Initiative for Chronic Obstructive Lung Disease (GOLD) guidelines (PFT showed an obstructive pattern with a ratio of forced expiratory volume in the first second (FEV1) to forced vital capacity (FVC) of less than 70% in post bronchodilator spirometry) [13].

Patients were excluded if they (1) had a comorbidity that could limit daily life, such as cancer, myocardial infarction, brain hemorrhage, and brain infarction, (2) were chronically taking oral steroids or immunosuppressive drugs because of rheumatology disease or organ transplants, (3) were taking antibiotics due to an acute infection, (4) if they did not do PFT, or (5) if FeNO was performed during hospitalization.

### 2.3. Statistical Analysis

Data were analyzed using SPSS 20 software (SPSS for Windows, IBM Corporation, Armonk, NY, USA). Data were presented as average ± standard deviation (SD). The correlation coefficients between FeNO and PM_10_ were analyzed by a Pearson correlation analysis. To identify the most correlated times, we analyzed the correlation between FeNO and PM_10_ daily averages from the day of visit to 4 days before visit. All FeNO tests were performed on the day of the outpatient visit. In addition, we performed a multivariate linear regression analysis that included various factors affecting FeNO. We adjusted patient age, sex, history of respiratory disease, pulmonary related treatments before the visit, humidity, and temperature in a multivariate analysis. In the multivariate analysis, *B* was the regression coefficient, and the positive sign of the regression coefficient meant that the variables were positively associated. The trend lines were obtained by a linear model, using the equation *Y* = *b*0 + (*b*1 × *t*). “*b*1” is a slope of the regression line, and “*b*0” is an intercept of the regression line with the *Y*-axis. An additional sub-analysis was conducted by dividing the group according to history of respiratory disease and pulmonary related treatment. In our sub-analysis, “no history of respiratory disease” was defined as the group without asthma, COPD, allergic rhinitis, and no obstructive or restrictive pattern on the PFT. And, “no pulmonary medication” was defined as the group that did not use any medication or inhaler. *Pvalues* less than 0.05 were defined as statistically significant.

## 3. Results and Discussion

### 3.1. Baseline Characteristic

According to the exclusion criteria, 1,574 FeNO results and 1,439 patients were included (Table 1). The average age was 48.3 ± 16.1 years. Men were 43.7 percent and women 56.3 percent of the patients. The asthma patients were 23.3 percent, the allergic rhinitis patients were 15.4 percent, and the COPD patients were 3.0 percent. 19.3 percent of events were using one or more pulmonary-related medications. 70.5 percent of events showed normal PFT findings. And the average value of PM10 was 47.3 ± 25.2 *μ*g/m^3^, and the average value of FeNO was 31.9 ± 30.8 ppb.

### 3.2. Correlation between PM_10_ and FeNO

**Figure 1:**
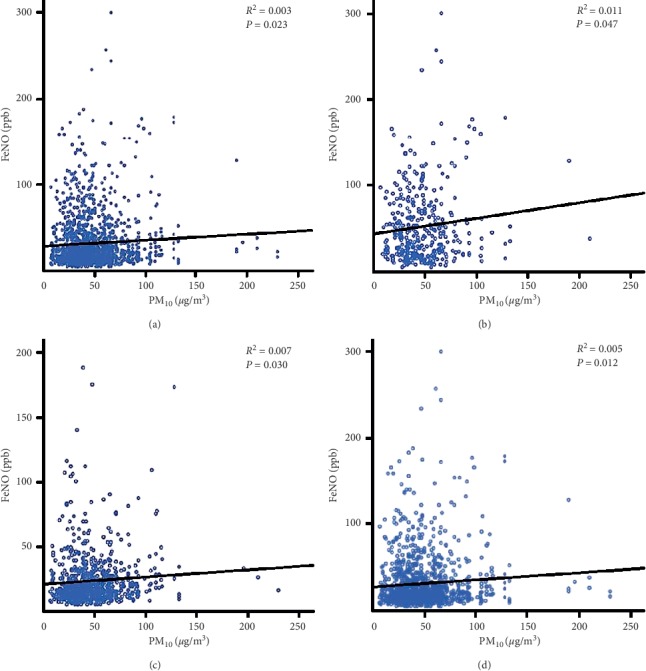
Trend line according to PM_10_ and FeNO. (a) All. (b) Asthma. (c) No history of respiratory disease. (d) No pulmonary medication. PM_10_ values were based on two days before hospital visit. PM_10_, particulate matter with a diameter of less than 10 *μ*m; FeNO, fractional exhaled nitric oxide.

In the Pearson correlation, FeNO positively correlated with PM_10_ at the day of the hospital visit (Pearson correlation coefficient = 0.061; *P* value = 0.016). And, FeNO also positively correlated with PM_10_ at two days before hospital visit (Pearson correlation coefficient = 0.057; *P* value = 0.023) (Figure 1(a)). There were no statistically significant correlations with other days. In the multivariate linear regression analysis, there was a positive correlation between FeNO and PM_10_, which was measured two days before the hospital visit (*B* = 0.051, *P* value = 0.026). If the PM_10_ increased by 100 *μ*g/m^3^, the FeNO value was expected to rise to 5.1 ppb. And, there was no statistically significant correlation with other days, including PM_10_ at the day of hospital visit (Table 2).

### 3.3. Subgroup Analysis

We performed the subgroup analysis according to the patient's history of respiratory disease and the pulmonary-related medication before the visit. And, the PM_10_ value two days before the hospital visit was used as the reference value because it was statistically significant in the multivariate analysis. PM_10_ results were similar among the subgroups. FeNO values were highest in the asthma group and lowest in the “no history of respiratory disease” group. In the Pearson correlation, the asthma group (Pearson correlation coefficient = 0.104; *P* value = 0.047), the “no history of respiratory disease” group (Pearson correlation coefficient = 0.081; *P* value = 0.030) and the “no pulmonary medication” group (Pearson correlation coefficient = 0.070; *P* value = 0.012) showed a positive correlation with FeNO (Figures 1(b)–1(d)). In the multivariate linear regression analysis, there was a positive correlation between FeNO and PM_10_ in the “no history of respiratory disease” group (*B* = 0.083, *P* value = 0.024) and the “no pulmonary medication” group (*B* = 0.053, *P* value = 0.035). And, there was no statistically significant correlation with the other groups, including the asthma group (Table 3).

## 4. Discussion

This study is the first to demonstrate the correlation between PM_10_ and FeNO in Korea. A total of 1,574 events were included, and various factors that could affect FeNO were investigated. In addition to a univariate analysis, we performed a multivariate analysis to adjust various factors that could affect FeNO. Through the subgroup analysis, we confirmed the same results under various conditions. As such, we confirmed a positive correlation between PM_10_ and FeNO.

PM is generated from automobile exhaust and from construction and various industries, and it is a complex containing heavy metals and toxic chemicals. It is generally known that PM is composed of sulfate, nitrate, carbon, and black dust. PM_10_ means a PM with a diameter of less than 10 *μ*m, which is about one-fifth of that of human hair. PM stays in the atmosphere and is absorbed into the body through the skin, eyes, and the part of the respiratory tract exposed to the outside [14, 15]. PM can act as an irritant or allergen at the primary contact site and causes local adverse effects. In addition, PM causes abnormal inflammation and anticoagulation in the body [16, 17]. The airway, which is the primary exposed organ, is considered to have higher adverse effects from PM than other organs [18]. Even in healthy people, exposure to PM can lead to abnormal inflammatory responses in the airways.

PM is associated with the development and aggravation of respiratory diseases, such as asthma and COPD. In addition to the respiratory system, PM can cause disease in all organs of the body, including myocardial infarction, brain stroke, atopic dermatitis, and allergic rhinitis. On high PM days, healthy people also complain of nonspecific symptoms, such as runny nose, sputum, cough, and itching. Most of the PM-related studies so far have primarily focused on patients, but studies are also needed to analyze its effects on healthy people.

Nitric oxide (NO) is a biological mediator produced in human lungs [19]. The NO produced in the lungs is released outside during exhalation [20, 21]. NO is known to act as a vasodilator, bronchodilator, neurotransmitter, and inflammatory mediator in the airway [22–24]. Recently, NO was recognized as an important key to understanding lung biology and the pathophysiology of airway diseases. When inflammation occurs in the airway, nitric oxide synthase is upregulated and NO is generated in airway epithelial cells [25, 26]. The FeNO test has been increasingly used because it can easily and noninvasively measure NO. FeNO is also known to be associated with various interleukins, cytokines, and sputum eosinophilia [27, 28]. Therefore, FeNO is considered a test reflecting airway inflammation and airway hyperresponsiveness [29–31].

FeNO has a broad clinical use. The National Institute for Health and Care Excellence (NICE) guideline says that FeNO can be used for asthma diagnosis [32]. Other researches recommend that FeNO is useful as a reference for diagnosing asthma [33]. In particular, FeNO is more useful for young children who have difficulty performing PFT [34]. Some studies suggest that FeNO is also useful for screening high-risk groups for asthma [35]. FeNO is also useful for predicting ICS response [36]. Currently, medication control is symptom dependent for the treatment of asthma. FeNO can be used as an objective indicator for medication control [37].

Few studies have focused on the correlation between FeNO and PM [38]. Most of the previous studies were small cases or were limited to children [39]. In addition, most of the studies were conducted on asthma patients [40]. Our study was the first to include a large number of patients and healthy people.

Our research had some limitations. First, we did not adjust the height, weight, smoking history, nitric oxide, diet, house air quality, seasonality, and weather conditions in the multivariate analysis. FeNO is affected by various factors, for example, drugs, history of respiratory disease, gender, age, height, weight, smoking history, and nitric oxide level in atmosphere. Data on height, weight, and smoking history were not accurate and could not be analyzed. But, regarding seasonality and weather conditions, we included humidity and temperature. Secondly, the degree of exposure of PM may be slightly different. The degree of exposure may vary depending on the level of activity and location. To compensate for this, we excluded inpatients. Also, we excluded patients with diseases in which physical activity could be limited. Third, the differences in medication dosages and compliance were not corrected. In cases of patients with antihistamines, ICS, and intranasal corticosteroids, there were those with differences in the components and dosages of the drugs. There may also be differences in the proficiency and compliance with the inhaler. Fourth, we did not have big data from repeated comparisons of the same person. If repeated tests were to be conducted on the same person, a more stringent comparison would be possible. In our study, 115 subjects were repeatedly examined. A positive correlation was also observed in the repeated test group. Fifth, our study design and statistical analysis are very simple, so there are a lot of confounding variables. To overcome this limitation, we included a large number of subjects. In addition, many factors were included in the meta-analysis.

## 5. Conclusions

We confirmed a positive correlation between PM_10_ and FeNO. Therefore, PM_10_ may cause airway inflammation. The same results were obtained in healthy persons as well as patients with respiratory conditions. Even healthy people may develop airway inflammation and respiratory symptoms caused by PM_10_. In addition, it should be considered that PM_10_ affects the FeNO value on days with very high PM_10_ value.

## Figures and Tables

**Table 1:** Baseline characteristics.

	Events
No. of outpatient clinic visits/patients	1,574/1,439
Age (years)^*∗*^	48.3 ± 16.1
Gender	
Male^†^	688 (43.7%)
Female^†^	886 (56.3%)
History of respiratory disease	
Asthma^†^	367 (23.3%)
Chronic obstructive pulmonary disease^†^	48 (3.0%)
Allergic rhinitis^†^	242 (15.4%)
Pulmonary-related medication before the visit	
INS^†^	82 (5.2%)
ICS^†^	204 (13.0%)
LABA or LAMA^†^	201 (12.8%)
LTRA^†^	153 (9.7%)
Antihistamine^†^	187 (11.9%)
Base-line spirometry after bronchodilation	
FEV1 (liters)^*∗*^	2.8 ± 0.8
FEV1 (% of predicted value)^*∗*^	87.5 ± 14.6
FVC (liters)^*∗*^	3.5 ± 1.0
FVC (% of predicted value)^*∗*^	90.1 ± 12.9
Ratio of FEV1 to FVC (%)^*∗*^	77.9 ± 10.1
Bronchodilator response (positive)^†^	273 (17.3%)
Asthma provocation test (positive)^†^	105 (6.7%)

INS, intranasal corticosteroids; ICS, inhaled corticosteroid; LABA, long-acting B agonist bronchodilator; LAMA, long-acting antimuscarinic agent bronchodilator; LTRA, leukotriene receptor antagonist; FEV1, forced expiratory volume in one second; FVC, forced vital capacity. ^*∗*^Numbers are presented as average ± standard deviation. ^†^Numbers are presented as *n* (%).

**Table 2:** Correlation analysis of PM_10_ and FeNO by the Pearson correlation and multivariate linear regression analysis (most correlated time).

PM_10_ measurement date	PM_10_ day average^*∗*^	Univariate		Multivariate	
		Correlation coefficient	*P* value	*B*	*P* value
The day of hospital visit	45.88 ± 20.24	0.061	0.016	0.043	0.064
One day before hospital visit	45.74 ± 21.22	0.046	0.067	0.034	0.145
Two days before hospital visit	47.33 ± 25.20	0.057	0.023	0.051	0.026
Three days before hospital visit	47.68 ± 25.06	0.049	0.053	0.040	0.083
Four days before hospital visit	47.88 ± 23.34	0.025	0.312	0.023	0.325

PM_10_, particulate matter with a diameter of less than 10 *μ*m; FeNO, fractional exhaled nitric oxide. *B* is the regression coefficient, and the positive sign of regression coefficient means that the variables are positively associated. The multivariate linear regression analysis is adjusted for age, sex, previous history of respiratory disease (asthma, allergic rhinitis, and chronic obstructive pulmonary disease), pulmonary-related medication before the visit (antihistamine, intranasal corticosteroids, inhaled corticosteroids, and leukotriene receptor antagonist), humidity, and temperature. ^*∗*^Numbers are presented as the average ± standard deviation.

**Table 3:** Correlation analysis of PM_10_ and FeNO by the Pearson correlation and multivariate linear regression analysis according to subgroup.

Subgroup	Number of events	FeNO^*∗*^ (ppb)	PM_10_^*∗*^ ( *μ*g/m^3^)	Univariate		Multivariate	
				Correlation coefficient	*P* value	*B*	*P* value
History of respiratory disease							
Asthma^†^	367	52.3 ± 43.8	48.0 ± 25.7	0.104	0.047	0.093	0.072
Allergic rhinitis^†^	242	44.1 ± 37.0	47.8 ± 24.9	0.091	0.160	0.096	0.128
Chronic obstructive pulmonary disease^†^	48	33.9 ± 44.8	47.9 ± 22.7	−0.036	0.807	−0.058	0.708
No history of respiratory disease^†^	715	23.2 ± 19.8	47.5 ± 25.7	0.081	0.030	0.083	0.024

Pulmonary-related medication before the visit							
Pulmonary medication^‡^	309	36.3 ± 29.5	46.7 ± 21.2	−0.05	0.926	0.013	0.814
No pulmonary medication^‡^	1265	30.8 ± 31.1	47.5 ± 26.1	0.070	0.012	0.053	0.035

PM_10_, particulate matter with a diameter of less than 10 *μ*m; FeNO, fractional exhaled nitric oxide. *B* is the regression coefficient, and the positive sign of regression coefficient means that the variables are positively associated. PM_10_ values were based on two days before the hospital visit. ^*∗*^Numbers are presented as mean ± standard deviation. ^†^Multivariate linear regression analysis is adjusted for age, sex, pulmonary-related medication before the visit (antihistamine, intranasal corticosteroids, inhaled corticosteroids, and leukotriene receptor antagonist), humidity, and temperature. ^‡^Multivariate linear regression analysis is adjusted for age, sex, previous history of respiratory disease (asthma, allergic rhinitis, and chronic obstructive pulmonary disease), humidity, and temperature.

## Data Availability

The data used to support the findings of this study are available from the corresponding author upon request.
